# Associations Between the Neural Stress Response and Symptoms of Anxiety and Depression

**DOI:** 10.1002/jnr.70019

**Published:** 2025-01-16

**Authors:** Marina Giglberger, Hannah L. Peter, Gina‐Isabelle Henze, Christoph Bärtl, Julian Konzok, Peter Kirsch, Brigitte M. Kudielka, Ludwig Kreuzpointner, Stefan Wüst

**Affiliations:** ^1^ Department of Psychology University of Regensburg Regensburg Germany; ^2^ Research Division of Mind and Brain, Department of Psychiatry and Psychotherapy CCM, Charité‐Universitätsmedizin Berlin, Corporate Member of Freie Universität Berlin Humboldt‐Universität Zu Berlin, and Berlin Institute of Health Berlin Germany; ^3^ Department of Epidemiology and Preventive Medicine University of Regensburg Regensburg Germany; ^4^ Department of Clinical Psychology, Central Institute of Mental Health, Medical Faculty Mannheim University of Heidelberg Heidelberg Germany

**Keywords:** acute stress, amygdala, fMRI, Scan*STRESS*, striatum

## Abstract

Anxiety and depression disorders show high prevalence rates, and stress is a significant risk factor for both. However, studies investigating the interplay between anxiety, depression, and stress regulation in the brain are scarce. The present manuscript included 124 law students from the LawSTRESS project. Anxiety and depression symptoms were assessed using the Hospital Anxiety and Depression Scale (HADS), and psychosocial stress was induced with the imaging stress paradigm Scan*STRESS*. Anxiety, but not depression scores, were significantly related to neural stress responses in a striato‐limbic cluster. Moreover, relative to women, men showed stronger associations between anxiety scores and activation in striatal and temporal clusters. A bifactor model of the HADS suggested a general factor characterized by tension, nervousness, and cheerlessness, which was associated with activation changes in a similar but more circumscribed cluster than anxiety. In the LawSTRESS project, the HADS was assessed at five sampling points (1 year, 3 months, 1 week prior exam, 1 week, and 1 month thereafter), and thus an exploratory trajectory analysis could be performed. It confirmed the relationship between anxiety scores and striatal stress responses at baseline but revealed no predictive value of the neural measure across the sampling points. Our results suggest that—in healthy young participants—neural acute psychosocial stress responses in striato‐limbic structures are associated with anxiety, supporting the assumption that these regions are related to individual differences in vulnerability to stress‐related disorders. A correlation with depression scores could not be found, and possible explanations are discussed.

## Graphical abstract

Anxiety, but not depression scores, were significantly related to neural stress responses in a striato‐limbic cluster. Moreover, relative to women, men showed stronger associations between anxiety scores and activity in striatal and temporal clusters. Baseline neural stress responses could not predict the anxiety trajectory during a particular stress phase.

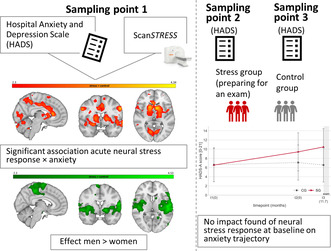

**Summary**

- It is known that stress is an important risk factor for anxiety and depression disorders. However, if and how acute stress responses in the brain are related to symptoms of anxiety and depression is not well understood.
- The present study found associations between striato‐limbic responses to psychosocial stress induction in an MRI scanner and anxiety scores in a young and healthy sample which might link interindividual differences in these regions to a certain vulnerability or resilience.
- Additionally, sex‐specific anxiety‐related activation patterns emphasize the importance to further investigate sex differences of the neural basis of mental disorders.

## 1 Introduction

In 2019, one in eight people worldwide was living with a mental disorder, with anxiety and depression disorders being the most common and women being more frequently affected than men (Seedat et al. 2009; Otten et al. 2021; GBD 2019 Mental Disorders Collaborators 2022). During the COVID‐19 pandemic, the number of affected people rose with an estimated increase of 25.6% and 27.6% for anxiety and major depressive disorders (MDD), respectively (Santomauro et al. 2021). Stressful life events and the exposure to chronic stress have been shown to be significant risk factors for both the onset and maintenance of anxiety and depression disorders (Hammen 2005; Chrousos 2009; Monroe, Anderson, and Harkness 2019). Since the brain is the key organ orchestrating the interpretation of external and internal stimuli as well as complex psychobiological stress responses, it appears crucial to investigate individual differences in neural stress processing to further elucidate the stress–disease relationship (De Kloet, Joëls, and Holsboer 2005; McEwen 2007).

Using functional magnetic resonance imaging (fMRI) in healthy participants, striato‐limbic and frontal activation changes in response to acute psychosocial stress exposure have been repeatedly reported (Noack et al. 2019; Henze et al. 2020, 2021; Berretz et al. 2021; Qiu et al. 2022). These regions are also known to be activated differently in individuals with anxiety disorders or depression. For instance, emotional stimulation, like the presentation of emotional faces or words, revealed altered activations in striato‐limbic structures including the amygdala, insula, nucleus (ncl.) caudatus, and anterior cingulate cortex (ACC) in patients with anxiety disorders such as post‐traumatic stress disorder, social anxiety disorder, or specific phobia (Etkin and Wager 2007; Bruehl et al. 2014). MDD patients showed, compared to healthy controls, mostly increased baseline activity during rest in striatal and limbic regions and altered neural responses to emotional tasks (Hamilton et al. 2012; Palmer et al. 2015; Gong et al. 2020).

Only recently, first studies investigated neural responses to psychosocial stress in clinical samples. In MDD as well as in anxiety disorder patients, activation patterns differing from those in healthy control participants have been predominantly found in limbic and striato‐prefrontal regions (Boehme et al. 2014; Ming et al. 2017; Dong et al. 2022). For instance, in patients with social anxiety disorders relative to healthy controls, increased activation of the insula and decreased activation of the ventral striatum could be observed during a stress anticipation task (Boehme et al. 2014).

Studies in patient samples are undoubtedly very important. However, to identify risk factors and to trace potentially relevant temporal trajectories, they should be complimented by studies in healthy individuals with varying levels of anxiety and depression. To date, only a few studies investigated associations between anxiety or depression and acute neural stress responses in healthy participants. Dedovic et al. (2014) used a modified version of the Montreal Imaging Stress Task (MIST) and found differences between students with subclinical depression and a healthy control group. Overall, activation changes were rather similar in both groups, but depression scores of the entire sample were positively related with changes in deactivation within the subgenual ACC. In a recent study using the MIST in adolescents, trait anxiety was associated with increased hippocampal and ventral striatal and decreased putamen activation (Corr et al. 2021). Moreover, trait anxiety in young, healthy adults was found to be positively associated with activation in medial prefrontal cortex, posterior cingulate cortex (PCC), and insula during the MIST stress condition (Wheelock et al. 2016).

Another useful imaging stress paradigm is Scan*STRESS* (Streit et al. 2014; Henze et al. 2020). It induces significant cortisol, heart rate, and subjective stress responses, and it alters activation in stress‐related neural networks (Streit et al. 2014; Henze et al. 2020; Dimitrov‐Discher et al. 2022; Liu et al. 2023). Recently, this paradigm has been used to study the effect of trait anxiety on resting state functional connectivity after stress exposure. The authors reported a positive relationship between trait anxiety and post‐stress connectivity between right amygdala and pregenual ACC/ventromedial PFC (Nanni‐Zepeda et al. 2022).

The present study explored the association between anxiety, depression, and acute neural stress responses cross‐sectionally at baseline in a subsample of the longitudinal LawSTRESS project (Giglberger et al. 2022; for the protocol see https://epub.uni‐regensburg.de/51920/). Participants in this study were young and healthy law students. In addition to a comprehensive baseline assessment (to which the analyses reported in the present paper primarily refer), five further measurement points were conducted over a period of more than 1 year. We could recently show that momentary perceived stress in daily life in students experiencing a chronic stress phase lasting several months could be significantly predicted by neural acute stress responses in (pre)limbic regions assessed at baseline (Giglberger et al. 2023).

To assess anxiety and depression scores, the Hospital Anxiety and Depression Scale (HADS; Zigmond and Snaith 1983) has been employed in the LawSTRESS project. The HADS is an established and globally used brief self‐rating scale with a high acceptance rate. Its design as a questionnaire with two subscales was supported by initial studies investigating the factor structure of the HADS (Bjelland et al. 2002). The German manual as well suggests a two‐factor solution that closely aligns with the originally proposed two subscales (Herrmann‐Lingen, Buss, and Snaith 2011). However, the authors admitted that in different populations, other factor structures, e.g., uni‐ or three‐dimensional solutions were found to have a better fit (Bjelland et al. 2002). In the following years, the uncertainty about the bidimensional structure has grown. Conceptually, anxiety and depression are highly associated, and their symptoms strongly overlap, making their differentiation often difficult (Kessler et al. 2015; Kalin 2020).

The aim of the present study was to investigate whether neural responses to stress are associated with anxiety or depression in a young and healthy sample. Based on research in patient samples (Boehme et al. 2014; Ming et al. 2017; Dong et al. 2022) and samples at risk (Dedovic et al. 2014; Wheelock et al. 2016; Corr et al. 2021), we hypothesized that HADS anxiety and depression scores are positively related to the overall neural stress response, especially in striato‐limbic regions. In view of the unclear factor structure of the HADS, a confirmatory factor analysis was computed in our sample in order to test—if appropriate—an alternative factor structure for its association with neural stress responses as well. Lastly, taking advantage of our longitudinal study design in an exploratory analysis, we assessed the predictive value of our neural results on the trajectory of anxiety and depression scores.

## 2 Methods

### 2.1 Sample

We included two groups of law students in our study, and we assumed that they did not differ in our dependent variables at baseline. One half were law students in preparation for the first state examination (stress group, SG) which in Germany is a long and challenging exam period that is perceived as very stressful by the vast majority of candidates (Rabkow et al. 2020; Giglberger et al. 2022). The other half were law students in the mid‐phase of their study program forming the control group (CG). Participants were recruited via flyers, social media, and presentations in universities as well as commercial law school courses and lectures. The current manuscript reports on data from a subsample of the LawSTRESS project comprising all 124 participants who underwent MRI (for a description of the entire study sample, see Giglberger et al. 2022). Of these, 13 were excluded due to pronounced motion artifacts despite motion correction (i.e., absolute movement > 3 mm during at least one run; *n* = 12) or due to poor image acquisition (*n* = 1), resulting in 111 participants (SG: 56, women: 33, hormonal contraceptives: 27, age: 21.16 ± 2.05; CG: 55; women: 37, hormonal contraceptives: 28, age: 22.75 ± 1.61). The gender distribution did not differ between the groups (*χ* ^2^(1) = 0.83, *p* = 0.362). Women not using hormonal contraceptives were scheduled for the MRI sessions during the luteal phase of the menstrual cycle (Wolfram, Bellingrath, and Kudielka 2011) determined by a chromatographic urinary ovulation test kit (gabmed GmbH, Köln, Germany). Individuals who met any of the following self‐reported criteria were excluded: Current psychiatric, neurological, or endocrine disorders, treatment with psychotropic medications or any other medication affecting central nervous system or endocrine functions, and regular nightshift work or MRI‐scanner contraindications (e.g., pregnancy and metal parts in the body).

The study was conducted in accordance with the Declaration of Helsinki, and it was approved by the local ethics committee of the University of Regensburg. All participants provided written informed consent and received monetary compensation as well as individualized feedback on their study results.

### 2.2 General Procedure

**FIGURE 1:**
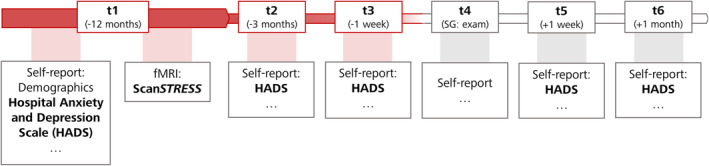
Timing of data collection. Red color indicates the aspects important for the current manuscript. For an overview of the entire study procedure of the LawSTRESS project, see https://epub.uni‐regensburg.de/51920/.

Except for the analysis described in Section 2.9., only variables that were collected at baseline were relevant for the present paper (see Figure 1). Consequently, the division into stress and control group was not relevant for the present main analysis. A detailed overall description of the LawSTRESS project can be found elsewhere (https://epub.uni‐regensburg.de/51920/). Briefly, the protocol comprised six sampling points (t1–t6) over 13 months. For SG, t1 was 1 year, t2 3 months, and t3 1 week prior exam. T4 was at the middle of the 8‐days exam period, t5 1 week, and t6 1 month thereafter (Figure 1). The same procedure, except the participation in the exam at t4, applied to the CG. Data collection was carried out in different waves and lasted from March 2018 until April 2021. At t1, an online questionnaire battery was submitted via SoSci Survey (https://www.soscisurvey.de; Leiner 2021) to assess baseline data, psychometrics (including the HADS to assess anxiety and depression), physical health, health behavior, and university studies‐related variables. Some of these data (including HADS scores) were collected again at t2, t3, t5, and t6. At baseline, an MRI scanning session (including the stress paradigm) took place for the MRI subsample.

### 2.3 Assessment of Anxiety and Depression

The HADS (Zigmond and Snaith 1983) is a brief (14 items) self‐assessment scale. It was originally designed as a screening questionnaire for clinically significant anxiety and depression in nonpsychiatric hospital settings, but it can also be used in nonclinical samples, and the manual includes norm values from both clinical samples and samples from the general population (Herrmann‐Lingen, Buss, and Snaith 2011). The anxiety (HADS‐A) and depression subscales (HADS‐D) each comprise seven items with a four‐point Likert scale (0–3). The total score for each subscale is calculated by adding the raw scores (range 0–21). A score ≥ 8 is considered as borderline and ≥ 11 as clinically relevant for both subscales (Zigmond and Snaith 1983). According to Bjelland et al. (2002), the cutoff of 8 or above provides an optimal balance between specificity and sensitivity (both around 0.80 for HADS‐A and HADS‐D). Since its development in 1983, the HADS was not only used in different medical settings (e.g., cardiology or oncology) but also in large samples of the general population (e.g., Breeman et al. 2015; Djukanovic, Carlsson, and Årestedt 2017). Its international distribution—it has now been translated into more than 65 different languages—makes it a frequently used instrument worldwide. In the present study, the HADS was administered to all participants as part of an online questionnaire battery submitted via SoSci Survey (https://www.soscisurvey.de/; Leiner 2021) and had to be filled out at least 1 day before the MRI measurement and at the sampling points t2, t3, t5, and t6. While five sampling points over 13 months do not allow a high‐resolution course analysis, they can serve as a data basis for an exploratory analysis of anxiety and depression score trajectories.

### 2.4 Scan*STRESS*

#### 2.4.1 Protocol

Scan*STRESS* (Streit et al. 2014; detailed description of the protocol in Henze et al. 2020) is a stress induction paradigm conceptualized for fMRI conditions primarily based on the psychological components uncontrollability and social‐evaluative threat (Dickerson and Kemeny 2004). The paradigm is composed of a block design with two runs containing two conditions (stress vs. control) each and a total duration of 24 min. During the stress blocks, the participants are instructed to perform visually presented computational and mental rotation tasks quickly and accurately via keystroke. Task speed and difficulty are adapted to the participant's performance, thereby forcing failure. After trials and between runs, two observers visible to the participants via video stream give standardized negative feedback on working speed and error frequency. During control blocks, simple figure‐ and number‐matching tasks have to be performed in the absence of time pressure and negative feedback. In the present study, test sessions were scheduled between 1:00 and 5:00 p.m.

#### 2.4.2 Salivary Cortisol, Heart Rate, and Subjective Stress

Saliva samples for later cortisol assessment, heart rate, and emotional reactivity ratings have been repeatedly obtained throughout the test session. In the present analysis, these variables served as a manipulation check to document the occurrence of acute psychoendocrine stress responses. A total of 10 saliva samples was collected—3 before the start of the paradigm (at −75, −15, and −1 min), 1 sample during the paradigm between the two Scan*STRESS* blocks (+15 min), sample immediately after the end of exposure (+30 min) as well as at minutes +50, +65, +80, +95, and +110. At the same sampling points, the participant's mood was surveyed using the German version of the Positive and Negative Affect Schedule (PANAS; Watson, Clark, and Tellegen 1988; Krohne et al. 1996). Due to standardization, the entire PANAS was presented, but only the negative affect (NA) was evaluated. Saliva samples were assayed in duplicate using a time‐resolved fluorescence immunoassay with fluorometric end‐point detection (DELFIA; Dressendörfer et al. 1992) at the biochemical laboratory at the University of Trier. The intra‐assay coefficient of variation was between 4.0% and 6.7%, and inter‐assay coefficients of variation were between 7.1% and 9.0%. An MRI‐compatible finger oximeter (Nonin Medical, Model 7500 FO, Minnesota, USA) recorded the heart rate during Scan*STRESS* with a sampling rate of the highest heartbeat within 4 s.

### 2.5 Statistical Analysis of Salivary Cortisol, Heart Rate, and Subjective Stress

Data were analyzed using R v4.0.3 with the package rstatix (Kassambara and Alboukadel 2021). All models were estimated with Maximum Likelihood, and the significance level was set at *α* = 0.05. For the acute stress response, log‐transformed cortisol values (nmol/L) and PANAS NA scores served as within‐subject factors in separate repeated measures analyses of variance (rANOVA). Participants with a cortisol increase of at least 1.5 nmol/L between the individual pre‐stress level (sample ‐1 min) and the individual peak (highest level of the three samples after the end of exposure) were defined as cortisol responders (Miller et al. 2013). Regarding heart rate, an rANOVA for each run was computed. Greenhouse–Geisser corrections were applied where appropriate and only adjusted results are reported.

### 2.6 fMRI Data Acquisition

Participants were scanned in a Siemens MAGNETOM Prisma 3T MRI scanner (Siemens Healthcare, Erlangen, Germany) equipped with a 64‐channel head coil. Functional images were acquired with a blood‐oxygenation‐level‐dependent (BOLD) gradient echo‐planar imaging (EPI) sequence covering 37 axial slices (3 mm thick, 1 mm gap, voxel size = 3 mm isotropic, interleaved, TR = 2000 ms, TE = 30 ms, flip angle = 90°, matrix size = 64 × 64 mm^2^, FoV = 192 mm) and T1‐weighted volumes with a 3D magnetization‐prepared rapid gradient‐echo (MP‐RAGE) sequence (TR = 2400 ms, TE = 2.18 ms, flip angle = 9°, voxel size = 0.8 mm isotropic, distance factor = 50%). The complete MRI session included resting state and anatomical measurements after the stress paradigm, which are not reported in the present manuscript.

### 2.7 fMRI Preprocessing and Analysis

Data analyses were carried out with FSL 6.0 (Smith et al. 2004; Jenkinson et al. 2012) using FEAT version 6.0 (Woolrich et al. 2001, 2004). The first five EPI volumes were discarded to account for T1 saturation effects. The preprocessing procedure included MCFLIRT motion correction, slice timing correction, brain extraction using BET, spatial smoothing (Gaussian kernel, 8.0 mm FWHM), grand mean intensity normalization, and high‐pass temporal filtering with 120.0 s cutoff. FLIRT and FNIRT were used for intra‐individual coregistration and registration to the 2.0 mm MNI152 standard space (Henze 2021). Time‐series statistical analysis was performed using FILM. *Z* (Gaussianized *t/F*) statistic images were thresholded *a priori* nonparametrically using clusters determined by *z* > 3.1.

In a first step, general linear models (GLMs) were performed with six condition regressors (stress arithmetic subtraction, stress figure rotation, control numbers, control figures, and announcement of stress and control) and six motion regressors for each participant and each run (first level analysis, *z* > 3.1). Next, we analyzed mean responses for each participant over both runs (second level, *z* > 3.1). To study the overall group neural stress response, a mixed‐effects group analysis (third level, *z* > 3.1) was conducted (Henze et al. 2020). For the main task effects (stress > control; control > stress), corrections via familywise error (FWE) for multiple comparisons at a significance level of *p* < 0.025 (two‐tailed combined test FWE *p* < 0.050) were applied.

For our main hypotheses, we conducted two GLMs for the main task effect stress > control (third level, *z* > 2.3), one including HADS‐A (model 1) and one including HADS‐D (model 2) scores as continuous covariates (grand‐mean‐centered). Both included sex as dichotomous nuisance variable (Table S1). Since sex differences in anxiety and depression are prominent (e.g., Altemus, Sarvaiya, and Neill Epperson 2014) and some studies also indicate sex differences in the neural stress response (e.g., Goldfarb, Seo, and Sinha 2019; Henze et al. 2021), we performed additional unpaired two‐group analyses with continuous covariate interaction. Specifically, we calculated one model to assess the interaction between sex and HADS‐A (model 3) and another to evaluate the interaction between sex and HADS‐D (model 4). This approach enabled a comprehensive examination of the differences between men and women (men > women; women > men) concerning the relationship between HADS scores and the neural stress response (Table S2). Whole‐brain corrections were conducted with each contrast thresholded at FWE *p* < 0.025 (two‐tailed combined test FWE *p* < 0.050). For the analyses presented in this manuscript, no power analysis was conducted. However, we additionally applied the FSL tool Randomise to further test our results on a nonparametric basis and to enhance the robustness of our findings (*n* = 5000 iterations).

**FIGURE 2:**
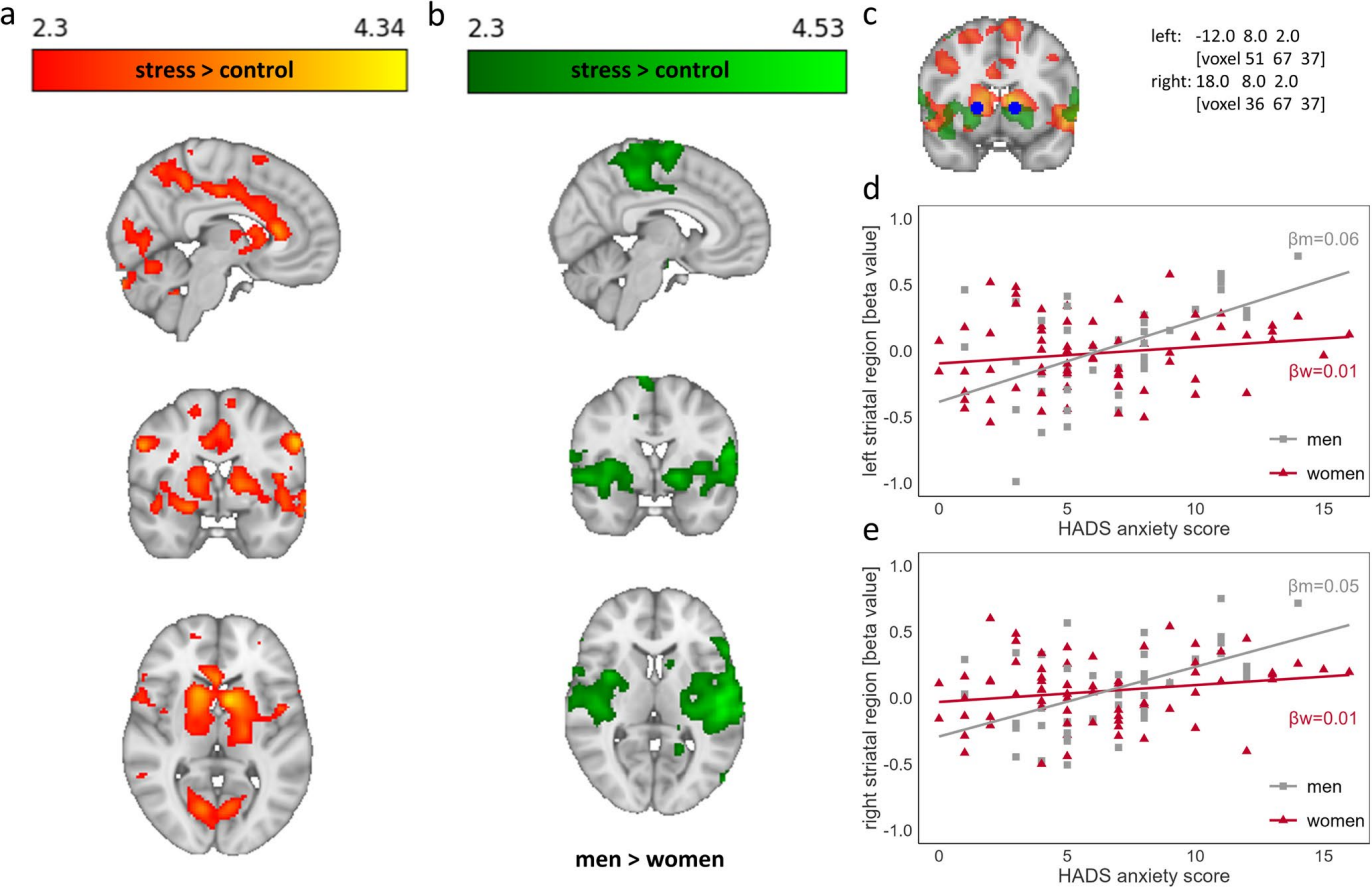
(a) Significant cluster derived from the analysis for the main task effect stress > control with HADS‐A scores (grand‐mean centered) as covariates and corrected for sex. (b) Sex‐specific clusters obtained by an unpaired two‐group analysis with continuous covariate interaction (HADS‐A, grand‐mean‐centered) describing a sex‐specific relationship (men > women) between anxiety and neural responses (stress > control). (c) Generated striatal mask around the overlapping peak region of the preceding whole‐brain analyses comprising parts of the ncl. caudatus, pallidum, and putamen (with MNI and voxel coordinates). (d) and (e) Sex‐specific correlations of HADS‐A scores with beta‐values of the main task effect stress > control in the left and right striatal ROI.

To further explore the relationship between stress‐responsive brain regions and anxiety or depression, a *post hoc* ROI analysis was performed utilizing fslmaths and featquery. Therefore, a binary 5 mm spherical mask around the overlapping peak region of the preceding (sex‐specific) HADS‐A whole‐brain analyses (comprising parts of the ncl. caudatus, pallidum, and putamen) for each hemisphere (left: 51 67 37; right: 36 67 37) was generated (Figure 2c). Mean beta‐values (extracted from second‐level analysis, i.e., from the individual stress response analysis) of the contrast stress > control were extracted for each participant and were subsequently entered into one‐way ANOVAs, with sex as the fixed factor and HADS‐A and HADS‐D as covariates.

### 2.8 Confirmatory Factor Analysis of the HADS

To explore the dimensionality of our HADS data, we conducted a confirmatory factor analysis (CFA) using the R lavaan package (Rosseel 2012). We performed one‐factor, two‐factor, and bi‐factor models (for further details, see Supporting Information Methods Section “Confirmatory factor analysis”). If a factor structure different from the original version is identified, we planned to repeat the same analytical procedure as utilized for the original HADS‐A and HADS‐D scores for the newly identified factor(s).

### 2.9 Exploratory Analyses: Predictive Value of Neural Responses on Anxiety and Depression Score Trajectories

To estimate a possible predictive value of our neural findings on anxiety and depression score trajectories until the exam (three assessments over 1 year, t1—t3), we performed generalized linear mixed models (time nested within participants) using the R glmmTMB package (Brooks et al. 2017). As only the participants in the SG were exposed to the “exam stress”, this analysis had to consider *study group* as relevant variable. The final models for both constructs contained the fixed‐effects *group* (0 = CG; 1 = SG), *time* in months as a linear trend, its interaction with *group*, and the covariate *sex* (0 = men; 1 = women). To account for dependencies in the data, random intercepts and slopes were estimated. In a next step, beta‐values of our precedingly described striatal *ROIs* (left and right) were included as main effect, in interaction with *group* and *time* trends and as three‐way interactions (*group* x *time* x *ROI*), since we were mainly interested in the ROI's impact on the group‐specific trajectories. Conditional R^2^ was calculated for the overall explained variance and marginal R^2^ for the variance explained by the fixed effects (Nakagawa and Schielzeth 2013).

## 3 Results

### 3.1 Hospital Anxiety and Depression Scale

In line with the findings of our overall sample (Giglberger et al. 2022), mean HADS‐A and HADS‐D scores at baseline (t1) were in the normal range (HADS‐A: 6.44 ± 3.60; HADS‐D: 3.41 ± 2.95), indicating that, on average, participants in our sample showed no/mild symptoms of anxiety and depression. Nevertheless, 35.1% (*n* = 39, women: 22) of the sample showed anxiety scores considered as borderline (scores ≥ 8 and ≤ 11) and 16.2% (*n* = 18, women: 10) exceeded the presumed clinically relevant score of 11. Regarding depression symptoms, 10.8% (*n* = 12, women: 9) of the participants reached the borderline score and 2.7% (*n* = 3, women: 3) a score considered as clinically relevant. Neither HADS‐A nor HADS‐D scores differed between SG and CG (HADS‐A: *t*(109) = −0.12, *p* = 0.905; HADS‐D: *t*(109) = −0.76, *p* = 0.450).

### 3.2 Associations of Anxiety and Depression Scores With Neural Responses

First, regarding acute stress reactions during Scan*STRESS*, cortisol changes and whole‐brain neural response patterns have been reported in Giglberger et al. (2023). Briefly, cortisol levels showed a significant mean rise following the stressor with a higher increase in men compared to women (responder rate: men: 73.8%; women: 37.0%). Furthermore, a distributed network of neural activations (stress > control) and deactivations (control > stress) comparable to prior studies could be found in response to stress exposure (e.g., Henze et al. 2020; for peak voxels, see Table S3). PANAS NA ratings rose significantly during the exposure to Scan*STRESS* (*F*_3.58, 386.69_ = 55.27, *p* < 0.001, *η*_p_^2^ = 0.34), and the heart rate was significantly higher during stress compared to control blocks in both runs (run1: *F*_2.41, 238.22_ = 135.69, *p* < 0.001, *η* ^2^ = 0.58; run2: *F*_2.07, 209.51_ = 181.07, *p* < 0.001, *η* ^2^ = 0.64).

To analyze the association of anxiety and depression with the neural stress reaction, individual HADS‐A (model 1) and HADS‐D (model 2) scores (both grand‐mean‐centered) were used as covariates in separate models (FWE‐corrected *p* < 0.050). Additionally, sex was entered as a covariate.

Adding HADS‐A scores significantly predicted neural responses (stress > control) in one cluster including ncl. caudatus, thalamus, putamen, parahippocampal gyrus, and insula (Figure 2a; peak voxels can be found in Table S4). Moreover, an unpaired two‐group analysis with HADS‐A scores as a continuous covariate (model 3) revealed a significant sex difference (men > women) in striatal and temporal clusters comprising superior temporal gyrus, ncl. caudatus, putamen, middle cingulate gyrus, and insula (Figure 2b; for peak voxels, see Table S5). Both results were also tested nonparametrically with Randomise (*n* = 5000 iterations), and they did not change (model 1: *z*_max_ = 4.51; model 3: *z*_max_ = 4.71). Illustrative *post hoc* ROI analyses for the chosen striatal region (Figure 2c; region of overlap between the effects of model 1 and model 2) supported these findings by showing a significant main effect for HADS‐A scores (left ROI: *F*_1,107_ = 12.50, *p* < 0.001, *η*_p_^2^ = 0.10; right ROI: *F*_1,107_ = 12.90, *p* < 0.001, *η*_p_^2^ = 0.10; both survive Benjamini–Hochberg correction) and a significant sex × HADS‐A interaction (left ROI: *F*_1,107_ = 8.60, *p* = 0.004, *η*_p_^2^ = 0.07; right ROI: *F*_1,107_ = 7.14, *p* = 0.009, *η*_p_^2^ = 0.06; both survive Benjamini–Hochberg correction) but no main effect for sex (left ROI: *F*_1,107_ = 0.78, *p* = 0.378; right ROI: *F*_1,107_ = 0.13, *p* = 0.724, Figure 2d,e). A cluster with higher task‐specific activation for women than men could not be detected. Entering depression scores to the neural analysis revealed no significant activations or deactivations associated with depression (model 2) nor a sex × depression interaction (model 4).

### 3.3 Confirmatory Factor Analysis of the HADS

The CFA revealed the best model fit for a bifactor model with 12 items (6 items each from the original anxiety and depression scale). Highest factor loadings on the general factor (HADS‐G) could be observed for items indicating frequent states of feeling tensed, nervous, and cheerless. Reliability indices supported the use of the HADS items in a composite total score (general factor: ω_H_ = 0.77, anxiety: ω_HA_ = 0.21, depression: ω_HD_ = 0.12). For a detailed description of the CFA results, see Supporting Information Results Section “Factor structure of the HADS”.

### 3.4 Associations of the HADS General Factor With Neural Responses

**FIGURE 3:**
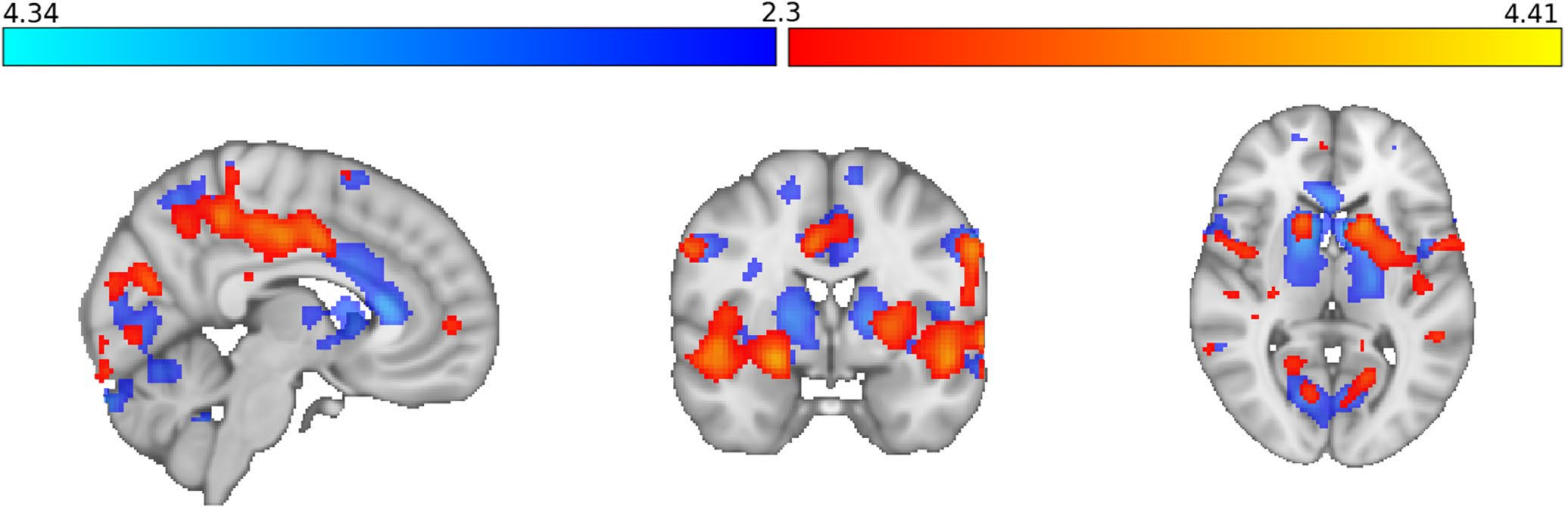
Significant clusters derived from the analysis for the main task effect stress > control (red to yellow) with the HADS general factor (composite score) as a continuous covariate corrected for sex compared with the clusters from the analysis with the anxiety scores (blue).

Taking the results of the CFA into account, we took a further step and exploratively analyzed the relationship between the discovered general factor and neural stress responses (FWE‐corrected *p* < 0.050). Adding this factor as a covariate revealed significant clusters (stress > control) including precuneus, middle cingulate cortex, putamen, ncl. caudatus, and the right amygdala which incorporates an activation peak (Figure 3; peak voxels can be found in Table S7). The results were tested additionally nonparametrically using Randomise (*n* = 5000 iterations), and they did not change *z*_max_ = 4.47. An unpaired two‐group analysis with HADS‐G as a continuous covariate revealed no significant sex‐specific clusters.

### 3.5 Exploratory Analysis: Predictive Value of the Neural Response on Anxiety Score Trajectories

The fMRI analysis indicated no significant difference in the neural stress response (stress > control) between the SG and the CG at baseline. Regarding the trajectory of anxiety symptoms in our sample over 1 year, we could show the expected difference between the SG and CG participants over time (time × SG *b* = 0.04, *p* < 0.001) but not at baseline (SG *b* = 0.03, *p* = 0.801), which is similar to our results in the entire study sample (Giglberger et al. 2022). In the SG, a steep rise of anxiety scores until the exam was observable, whereas the CG stayed relatively stable (see Figure S2). Adding beta‐values of our ROIs (left and right) to separate models confirmed their association with HADS‐A scores at baseline (left_ROI: *b* = 0.61, *p* = 0.010; right_ROI: *b* = 0.80, *p* = 0.004) but could not reveal an impact on the trajectory (all *p*s ≥ 0.075; see Table S8 for all model parameters). Since no activation patterns were found to be associated with HADS‐D scores in our previous analyses, we refrained from further longitudinal analyses.

## 4 Discussion

The aim of the present analysis was to determine whether anxiety or depression scores in a young and healthy sample are associated with an altered response to acute psychosocial stress. Therefore, Scan*STRESS* was used as an acute stressor. In our study, an elevated mean heart rate during stress versus control blocks and a rise in negative affect during the task could be observed. Mean salivary cortisol levels—as previously reported (Giglberger et al. 2023)—did also increase significantly during stress exposure, with men exhibiting higher mean increases than women, an often reported sex difference in stress research (Kudielka and Kirschbaum 2005; Zänkert et al. 2019). Regarding neural activation changes, a distributed network of activations and deactivations was found similar to previous findings (Streit et al. 2014; Henze et al. 2020). The observed affective, heart rate, and cortisol responses support the assumption that the paradigm elicits indeed psychosocial stress and not mere task‐related mental load.

The rates of borderline and potentially clinically relevant HADS‐A (borderline: 35.1%; clinically relevant: 16.2%) as well as HADS‐D scores (borderline: 10.8%; clinically relevant: 2.7%) seem rather high in our sample. However, these frequencies are consistent with reports of higher prevalence rates of mental health problems in student cohorts compared to cohorts from the general population (Ibrahim et al. 2013; Burger et al. 2014). In a recent study with students from various disciplines, 33.8% reported depression symptoms and 40.2% anxiety symptoms (Dastan et al. 2023).

Regarding our main hypotheses, a significant relationship between anxiety scores and the neural stress response could be found. More specifically, the whole brain analysis with HADS‐A scores as additional covariate revealed a significant striato‐limbic cluster comprising, among others, ncl. caudatus, thalamus, putamen, parahippocampal gyrus, right amygdala, and insula. Limbic structures are known to be involved in reactivity to and processing of negative emotional stimuli (Phan et al. 2002; Kober et al. 2008). Furthermore, compared to healthy controls, patients with anxiety disorders were found to react to negative emotional situations with hyperactivity in limbic regions such as amygdala or insula (Etkin and Wager 2007). Striatal regions are not necessarily discussed as key structures for stress or anxiety processing. However, in a meta‐analysis, subregions of the striatum were found to be consistently activated by acute psychological stressors regardless of the used paradigm (Berretz et al. 2021). Additionally, the striatum plays an important role in reward sensitivity (Haber and Knutson 2010; Liu et al. 2011), which is altered in patient samples (Admon et al. 2015; Cremers et al. 2015) and prone to stress in healthy participants (Porcelli, Lewis, and Delgado 2012; Oei et al. 2014). Hence, the striatum may be a critical structure where stress and reward processing are integrated. The positive relationship between anxiety and stress‐related striatal activation might indicate a state of hypersensitivity during stress along with dysfunctional reward processing.

While sex differences in prevalence rates and severity of anxiety disorders have often been reported, their underlying neural basis is not well understood (Seedat et al. 2009; Höglund, Hakelind, and Nordin 2020; Merikangas and Almasy 2020; Otten et al. 2021). In the present study, we could detect a sex‐specific association between anxiety and neural responses in temporal and striatal clusters comprising superior temporal gyrus, ncl. caudatus, putamen, middle cingulate gyrus, and insula. In men compared to women, higher anxiety scores were related to more activation within these clusters. Additionally, *post hoc* ROI analyses for the chosen striatal regions confirmed the interaction of sex × HADS‐A scores. Regions of the temporal lobe were found to be involved in all kinds of anxiety disorders (Kent and Rauch 2003), and a meta‐analysis on sex differences in response to emotional stimuli exhibited greater activation for negative stimuli in men than women containing structures like right superior temporal gyrus, right precentral gyrus, left middle frontal gyrus, and insula (Stevens and Hamann 2012). Moreover, divergent striatal activation for men and women was shown for stress (Henze et al. 2021) and reward processing (Lighthall et al. 2012; Warthen et al. 2020), both possibly of significance for the development of anxiety disorders. Interestingly, a cluster with higher task‐specific activation for women than men could not be detected. Thus, our findings suggest a stronger involvement of a set of temporal and striatal regions in men only and further emphasize the importance of investigating neural sex differences in psychological disorder reasearch.

Regarding depression scores, we were not able to find significant associations with neural stress responses in our sample. To our knowledge, so far, there is only one other study which investigated the link between neural stress responses and depression in a sample showing subclinical levels of depression (Dedovic et al. 2014). The authors reported a positive relationship between depression scores in the entire sample and the subgenual ACC deactivation. Several differences could explain the divergent findings in our analysis. First, the samples were selected differently. While we recruited healthy law students from the general student population, Dedovic et al. (2014) specifically selected a healthy and a subclinically depressed group based on their Beck Depression Inventory scores (Beck, Steer, and Brown 1987). Thus, the variability of depression levels in our sample was possibly too small to find associations with the stress response (HADS‐D: 3.41 ± 2.95). This assumption is consistent with our finding that according to the HADS cutoff values, mean depression symptoms in our sample were less pronounced than anxiety symptoms. Second, the used stress paradigms, modified MIST and Scan*STRESS*, might be similar, but they are not the same. This can be illustrated, for instance, by the lack of significant mean cortisol stress responses following the MIST in the study by Dedovic et al. (2014) compared to a significant cortisol rise after stress exposure in the present study.

Since anxiety and depression disorders have a high comorbidity rate (Kessler et al. 2015; Kalin 2020) and the factor structure of the HADS varies across studies (Bjelland et al. 2002; Cosco et al. 2012), we exploratively examined the HADS factor structure in our sample. The best fit achieved a bifactor model with a general factor characterized by frequent states of feeling tensed, nervous, and cheerless, while the original two‐factor structure was on second place. This is in line with a meta‐analysis by Norton et al. (2013) which included 21 studies and supported a bifactor structure as well. Consequently, we investigated the relationship between the discovered general factor and the neural stress response and could find a similar but more circumscribed cluster as for the original anxiety scores. More circumscribed is meant in a sense of less white matter response related with mostly smaller (e.g., ACC, thalamus, or pallidum), but also larger (right amygdala) extents of responsiveness in individual structures. The association of right amygdala activation and anxiety scores fits perfectly in the already existing literature on fear and anxiety (Bruehl et al. 2014; Tovote, Fadok, and Lüthi 2015). However, the amygdala seems to respond more strongly to specific components of anxiety, as indicated by the pronounced association with the general factor, which is characterized by tension, nervousness, and cheerlessness. For the general factor, the activation peak of the cluster cannot only be localized in the right amygdala, but the extent of amygdala involvement is also increased compared to anxiety (HADS‐G: 113 voxels; HADS‐A: 64 voxels). On the other hand, also the anxiety score appears to incorporate information that could not be explained by the general factor (see structures like ACC, thalamus, or pallidum). Taking the CFA results into account, a certain worrying component of the HADS‐A score might lead to a heightened responsiveness in structures like the ACC or thalamus. Further studies might elucidate this specific component‐related difference.

Taking advantage of our longitudinal study design, we explored the predictive value of our neural results on the trajectory of anxiety symptoms. First, in line with our findings based on the entire LawSTRESS sample (Giglberger et al. 2022), we observed a significant group difference in anxiety over time with a mean increase of anxiety symptoms until the exam in the stress group, whereas the participants of the control group stayed relatively stable. Adding beta‐values of the striatal ROIs strengthened our whole‐brain findings by showing a significant association with anxiety at baseline (t1) but had—neither in the CG nor in the SG—an influence on its expression over time. In line with the “brain‐as‐predictor” approach (Berkman and Falk 2013), we think it is promising to search for associations between neural activation in laboratory contexts and longitudinally assessed, ecologically valid outcomes in everyday life to contribute to the understanding of human stress regulation. In a recent study, we could predict momentary perceived stress levels in daily life over 1 year by acute stress responses in (pre)limbic regions (Giglberger et al. 2023). However, we assume that our study design and our data were not optimal to predict future HADS anxiety scores. The statistical power of this exploratory analysis was limited as anxiety scores were assessed at only 3 times over 1 year until the exam. It could also be speculated that neural responses to anxiety‐specific paradigms, like the presentation of emotional pictures, might be conceptually closer to the perception of anxiety in everyday life than acute stress responses and, thus, more suitable in that specific case.

Our study has some further limitations that need to be addressed. First, including only law students was a well‐founded decision in the context of the longitudinal LawSTRESS project. However, this strategy limits the generalizability, and in this sense, we could have strengthened our results by recruiting a more diverse and general‐population‐based sample as supposed by the Research Domain Criteria (RDoC) framework (Insel et al. 2010). Moreover, in view of recent literature reporting smaller than expected effect sizes for associations between neural measures and complex human behavior, the number of participants in the present sample must be regarded as rather low (Marek et al. 2022). However, due to our specific study design (long‐lasting, clearly predictable future stress period and control group) this was not feasible. Second, anxiety and depression were assessed using self‐report scales only, and as some scores were assessed during a high‐stress period, these measurements were very likely influenced by the current stress. Additionally, as already mentioned, the factor structure of the HADS is not certainly clarified. Future studies might use questionnaires specifically developed for anxiety or depression. However, we chose the HADS because it asks for rather mild symptoms without physical indicators which was assumed to better differentiate in an otherwise healthy cohort. Moreover, since the HADS does not inquire, for example, about suicidal tendencies, its items are usually well accepted by the participants. Lastly, future studies may consider a second fMRI scan following a stressful period to assess not only psychometric variables over time but also potential neural alterations.

To conclude, anxiety but not depression scores in a young and healthy cohort were found to be significantly associated with stress responses in a striato‐limbic cluster giving rise to the assumption that interindividual differences in these regions may contribute to a certain vulnerability or resilience. The sex‐specific anxiety‐related activation pattern emphasizes the importance to further investigate sex differences of the neural basis of mental disorders. After detailed examination of the HADS factor structure, a general underlying factor characterized by frequent states of feeling tensed, nervous, and cheerless could also be linked to a similar but more circumscribed cluster indicating potential anxiety component‐related activation changes. A predictive value of acute stress responses in striatal ROIs on subsequent expression of anxiety symptoms during a chronic stress period could not be detected.

## Declaration of Transparency

The authors, reviewers and editors affirm that in accordance to the policies set by the *Journal of Neuroscience Research*, this manuscript presents an accurate and transparent account of the study being reported and that all critical details describing the methods and results are present.

## Author Contributions

M.G., H.L.P., P.K., B.M.K., and S.W. conceptualized the study. M.G., H.L.P., G.‐I.H., C.B., and J.K. conducted the experiment. G.‐I.H. provided support with fMRI analysis. L.K. provided support with setting up and interpreting the confirmatory factor analysis. M.G. conducted the final data analysis. M.G. and G.‐I.H. interpreted the data, and M.G. wrote the original manuscript. All authors revised the manuscript.

## Conflicts of Interest

The authors declare no conflicts of interest.

### Peer Review

The peer review history for this article is available at https://www.webofscience.com/api/gateway/wos/peer‐review/10.1002/jnr.70019.

## Supporting information


Supporting Information S1.

**Data S1.** Transparent Science Questionnaire for Authors

## Data Availability

The data that support the findings of this study are openly available in the publication server of the University of Regensburg at https://epub.uni‐regensburg.de/59523, doi: 10.5283/EPUB.59523.
