# Supplementary material for: Associations Between the Neural Stress Response and Symptoms of Anxiety and Depression
Source: J Neurosci Res. 2025 Jan 16;103(1):e70019. doi: 10.1002/jnr.70019 (PMC11737356; doi:10.1002/jnr.70019)
Supplement: Supplementary file 1 — Supporting Information S1. [file JNR-103-e70019-s002.docx]

SUPPLEMENTS: Associations between the neural stress response and symptoms of anxiety and depression

Marina Giglberger^1^, Hannah L. Peter^1^, Gina-Isabelle Henze^1,2^, Christoph Bärtl^1^, Julian Konzok^1,3^, Peter Kirsch^4^, Brigitte M. Kudielka^1^, Ludwig Kreuzpointner^1^, Stefan Wüst^1^*

^1^ Department of Psychology, University of Regensburg, Regensburg, Germany

^2^ Research Division of Mind and Brain, Department of Psychiatry and Psychotherapy CCM, Charité-Universitätsmedizin Berlin, Corporate Member of Freie Universität Berlin, Humboldt-Universität zu Berlin, and Berlin Institute of Health, Berlin, Germany

^3^ Department of Epidemiology and Preventive Medicine, University of Regensburg, Germany

^4^ Department of Clinical Psychology, Central Institute of Mental Health, Medical Faculty Mannheim, University of Heidelberg, Heidelberg, Germany

* Correspondence concerning this article should be addressed to:

Stefan Wüst, Department of Psychology, Universitätsstraße 31, 93053 Regensburg, Germany.

Phone: +49 (0)941 943 5646, E-Mail: stefan.wuest@ur.de

Keywords: fMRI, acute stress, amygdala, striatum, Scan*STRESS*

[1. Supplemental Methods 2](#_Toc158198875)

[1.1. Confirmatory factor analysis 2](#_Toc158198876)

[2. Supplemental Results 3](#_Toc158198877)

[2.1. Stress-induced whole brain activations and deactivations 3](#_Toc158198878)

[2.2. Associations of anxiety scores with neural responses 4](#_Toc158198879)

[2.3. Factor structure of the HADS 6](#_Toc158198880)

[2.4. Associations of the HADS general factor with neural stress responses 8](#_Toc158198881)

[2.5. Predictive value of neural responses on real-life anxiety 9](#_Toc158198882)

[References 11](#_Toc158198883)

# Supplemental Methods

## Parameters for the statistical analyses investigating the main hypotheses

Table S1. Statistical models for the investigation of the association between HADS-A (model 1) and HADS-D (model 2) with the neural stress response (tested in separate models).

| **Contrast** | **Women** | **Men** | **HADS-A/HADS-D score** |
| --- | --- | --- | --- |
| Anxiety/depression | 0 | 0 | 1 |

Table S2. Statistical models for the investigation of the sex-specific associations between HADS-A (model 3) and HADS-D (model 4) with the neural stress response (tested in separate models).

| **Contrast** | **Women** | **Men** | **HADS-A/HADS-D score women** | **HADS-A/HADS-D score men** |
| --- | --- | --- | --- | --- |
| Women > men | 0 | 0 | 1 | -1 |
| Men > women | 0 | 0 | -1 | 1 |
| Women | 0 | 0 | 1 | 0 |
| Men | 0 | 0 | 0 | 1 |

## Confirmatory factor analysis

Although numerous studies have already examined the underlying dimensionality of the HADS, findings are still inconsistent (Bjelland et al., 2002; Cosco et al., 2012). Therefore, we decided to conduct a confirmatory factor analysis (CFA) using the R lavaan package (Rosseel, 2012). The models were estimated with Maximum Likelihood and the significance level was set at α = .05. We evaluated the goodness-of-fit using the χ^2^/df ratio, comparative fit index (CFI), Tucker-Lewis index (TLI), the root mean square error of approximation (RMSEA), and the standardized root mean square residual (SRMR). A good fit index is considered to be close to or higher than a CFI and TLI score of .95 and close to or lower than a χ^2^/df ratio of 3, RMSEA score of .06 and SRMR score of .08 (Hu and Bentler, 1999). We performed one-factor, two-factor, and bi-factor models. In case of the decision for a bi-factor model, the relevance of the general and the specific factors was evaluated examining the hierarchical omega indices (ω_H_s). A good fit is indicated by a ω_H_ ≥ .70 for the general factor and ω_HS_ ≥ .30 for specific factors (Rodriguez et al., 2016).

# Supplemental Results

## Stress-induced whole brain activations and deactivations

Table S3. Activated and deactivated structures under psychosocial stress (two tailed combined FWE-corrected for whole brain, threshold < .050) including z- and p-values as well as the localization of peak voxels.

| **Brain structure** | | **statistics** | | | **MNI coordinates** | | |
| --- | --- | --- | --- | --- | --- | --- | --- |
|  |  | ***k*** | ***p*** | ***z*** | **X** | **Y** | **Z** |
| **Insula** | **left** | **100415** | **< .001** | **6.92** | **-32** | **16** | **6** |
|  | right |  |  | 6.83 | 30 | 30 | 8 |
| Superior frontal gyrus | right |  |  | 6.81 | 22 | -2 | 50 |
| Middle frontal gyrus | right |  |  | 6.80 | 30 | 4 | 48 |
| Lateral occipital cortex | left |  |  | 6.76 | -18 | -72 | 40 |
| Middle frontal gyrus | left |  |  | 6.73 | -30 | 2 | 54 |
| **Medial frontal cortex** | **right** | **31342** | **< .001** | **-7.26** | **2** | **40** | **-20** |
|  | left |  |  | -6.92 | -6 | 34 | -26 |
| Subcallosal cortex | left |  |  | -6.60 | -2 | 10 | -14 |
| Inferior frontal gyrus, orbital | right |  |  | -6.42 | 20 | 26 | -18 |
| Precuneus | right |  |  | -6.32 | 0 | -54 | 24 |
|  | left |  |  | -6.08 | 0 | -66 | 28 |
| **Angular gyrus** | **left** | **2361** | **.008** | **-6.79** | **-56** | **-66** | **34** |
|  | left |  |  | -6.65 | -54 | -64 | 42 |
|  | left |  |  | -6.59 | -54 | -72 | 34 |
| Inferior parietal gyrus | left |  |  | -5.30 | -40 | -80 | 46 |

*Note. k* = cluster size in voxels; MNI = Montreal Neurological Institute; global cluster maxima are in boldface.

## Associations of anxiety scores with neural responses

| **Brain structure** | | **statistics** | | | **MNI coordinates** | | |
| --- | --- | --- | --- | --- | --- | --- | --- |
|  |  | ***k*** | ***p*** | ***z*** | **X** | **Y** | **Z** |
| **Nucleus caudatus** | **left** | **25195** | **< .001** | **4.27** | **-8** | **10** | **8** |
| Superior temporal gyrus | left |  |  | 4.15 | -62 | 8 | -6 |
| Superior parietal gyrus | left |  |  | 4.14 | -20 | -46 | 50 |
| Precentral gyrus | left |  |  | 4.07 | -56 | -2 | 36 |
| Thalamus | left |  |  | 3.95 | -18 | -12 | 0 |
| Postcentral gyrus | left |  |  | 3.89 | -46 | -10 | 40 |

Table S4. Activated structures under psychosocial stress with anxiety scores as covariates and corrected for sex including z- and p-values as well as the localization of peak voxels.

*Note. k* = cluster size in voxels; MNI = Montreal Neurological Institute; global cluster maxima are in boldface.

| **Brain structure** | | **statistics** | | | **MNI coordinates** | | |
| --- | --- | --- | --- | --- | --- | --- | --- |
|  |  | ***k*** | ***p*** | ***z*** | **X** | **Y** | **Z** |
| **Superior temporal gyrus** | **left** | **7779** | **< .001** | **4.45** | **-52** | **-16** | **10** |
|  | left |  |  | 4.35 | -60 | -8 | 2 |
|  | left |  |  | 4.21 | -54 | -24 | 8 |
| Postcentral gyrus | left |  |  | 4.11 | -60 | 4 | 14 |
| Middle temporal gyrus | left |  |  | 3.98 | -44 | -24 | -10 |
| Pallidum | left |  |  | 3.96 | -18 | -2 | -4 |
| **Middle cingulate gyrus** | **right** | **5732** | **< .001** | **4.06** | **12** | **-28** | **40** |
|  | right |  |  | 3.92 | 10 | -36 | 48 |
| Postcentral gyrus | left |  |  | 3.52 | -24 | -34 | 72 |
| Supplementary motor area | right |  |  | 3.50 | 6 | -8 | 68 |
| Postcentral gyrus | left |  |  | 3.43 | -30 | -38 | 66 |
| Supplementary motor area | left |  |  | 3.40 | -12 | -8 | 72 |
| **Insula** | **right** | **4449** | **< .001** | **3.79** | **36** | **4** | **4** |
| Rolandic operculum | right |  |  | 3.70 | 62 | -14 | 14 |
| Superior temporal gyrus | right |  |  | 3.67 | 42 | -24 | 0 |
| Insula | right |  |  | 3.53 | 40 | 2 | -8 |
|  | right |  |  | 3.49 | 40 | 4 | -12 |
| Superior temporal gyrus | right |  |  | 3.48 | 54 | -4 | -4 |

Table S5. Activated structures under psychosocial stress for men > women with anxiety scores as covariates including z- and p-values as well as the localization of peak voxels.

*Note. k* = cluster size in voxels; MNI = Montreal Neurological Institute; global cluster maxima are in boldface.

## Factor structure of the HADS

No missing data were obtained since item responding was compulsory. For CFA, a one-factor model, a two-factor model, and a bifactor model was used. Due to a better model fit, one item of the anxiety (item HADS11) and one of the depression subscale (item HADS10) were excluded. The model fit indicated that the bifactor model with 12 items provided the best fit (Table S7). As presented in Figure S1, all the remaining items significantly loaded onto the factors. Reliability indices supported the use of the HADS items in a composite total score.

Table S6. Results of the confirmatory factor analysis.

| **Factor structure** | **χ ^2^** | **df** | **χ ^2^/df (*p*)** | **CFI** | **TLI** | **RMSEA** | **SRMR** |
| --- | --- | --- | --- | --- | --- | --- | --- |
| One factor (14 items) | 147.47 | 77 | 1.92 (*p* < .001) | .84 | .82 | .09 | .08 |
| One factor (12 items) | 109.26 | 54 | 2.02 (*p* < .001) | .86 | .83 | .10 | .08 |
| Two factors (14 items) | 140.80 | 76 | 1.85 (*p* < .001) | .86 | .83 | .09 | .08 |
| Two factors (12 items) | 104.25 | 53 | 1.97 (*p* < .001) | .87 | .84 | .09 | .08 |
| Bifactor (14 items) | 92.60 | 63 | 1.47 (*p* = .010) | .93 | .91 | .07 | .07 |
| Bifactor (12 items) | 61.17 | 42 | 1.46 (*p* = .030) | .95 | .93 | .06 | .06 |

*Figure S1.* Bifactor CFA model with 12 items. A = anxiety symptoms; D = depression symptoms; g = general factor.

## Associations of the HADS general factor with neural stress responses

| **Brain structure** | | **statistics** | | | **MNI coordinates** | | |
| --- | --- | --- | --- | --- | --- | --- | --- |
|  |  | ***k*** | ***p*** | ***z*** | **X** | **Y** | **Z** |
| **Precuneus** | **left** | **3763** | **< .001** | **3.92** | **-8** | **-48** | **52** |
| Middle cingulum | right |  |  | 3.80 | 16 | -40 | 40 |
| Precuneus | left |  |  | 3.49 | -18 | -46 | 50 |
| Middle cingulum | left |  |  | 3.42 | -14 | -40 | 40 |
|  | right |  |  | 3.37 | 6 | -18 | 40 |
|  | right |  |  | 3.31 | 8 | -26 | 44 |
| **Superior temporal pole** | **left** | **3478** | **< .001** | **4.03** | **-62** | **10** | **-6** |
| Superior temporal gyrus | left |  |  | 3.91 | -50 | 4 | -10 |
| Postcentral gyrus | left |  |  | 3.77 | -54 | -6 | 36 |
|  | left |  |  | 3.70 | -48 | -10 | 40 |
| Superior temporal gyrus | left |  |  | 3.49 | -64 | 4 | -2 |
| Nucleus caudatus | left |  |  | 3.45 | -14 | 12 | 6 |
| **Amygdala** | **right** | **2564** | **= .005** | **3.70** | **24** | **-2** | **-12** |
| Superior temporal gyrus | right |  |  | 3.46 | 52 | 4 | -10 |
|  | right |  |  | 3.44 | 46 | 0 | -8 |
| Putamen | right |  |  | 3.32 | 16 | 12 | -2 |
| Nucleus caudatus | right |  |  | 3.23 | 14 | 14 | 10 |
| Superior temporal gyrus | right |  |  | 3.18 | 58 | -20 | -4 |
| **Cuneus** | **right** | **2106** | **= .013** | **3.49** | **14** | **-70** | **22** |
| Calcarine sulcus | left |  |  | 3.34 | -6 | -70 | 16 |
| Lingual gyrus | right |  |  | 3.18 | 12 | -70 | 2 |
|  | left |  |  | 3.06 | -4 | -96 | -18 |
| Calcarine sulcus | left |  |  | 3.05 | 0 | -94 | -16 |
| Cuneus | left |  |  | 3.03 | -8 | -78 | 22 |

Table S7. Structures of the clusters of the main effect stress > control with HADS general factor scores as covariates corrected for sex including *z*- and *p*-values as well as the localization of the of peak voxels.

*Note. k* = cluster size in voxels; MNI = Montreal Neurological Institute; global cluster maxima are in boldface.

## Predictive value of neural responses on anxiety score trajectories


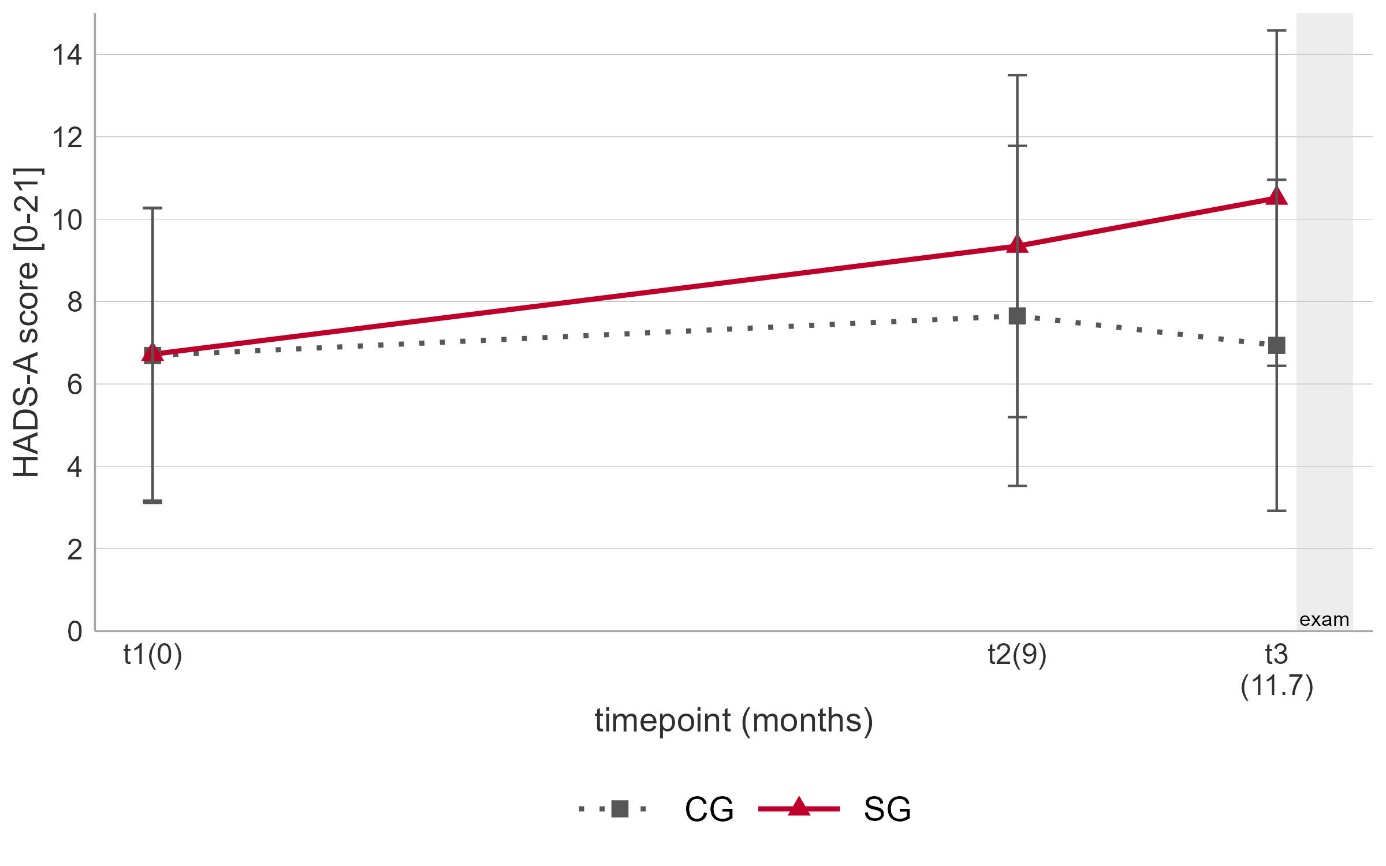


*Figure S2.* Time course of mean anxiety (HADS-A) scores (± SD) for stress (SG) and control group (CG).

Table S8. Parameter estimates for overall effects of the model with anxiety symptoms as dependent variable (basic.model) and the models with beta values of the left (left.ROI.model) and right (right.ROI.model) striatal ROI as predictor.

|  | **basic.model** | | | | **left.ROI.model** | | | | | **right.ROI.model** | | | | | |  |
| --- | --- | --- | --- | --- | --- | --- | --- | --- | --- | --- | --- | --- | --- | --- | --- | --- |
|  | **Estimates** | ***SE*** | | ***p*** | **Estimates** | | ***SE*** | | ***p*** | **Estimates** | | | ***SE*** | | ***p*** |  |
|  |  |  | |  |  | |  | |  |  | | |  | |  |  |
| Intercept | 1.85 | 0.10 | | **< .001** | 1.85 | | 0.09 | | **< .001** | 1.78 | | | 0.10 | | **< .001** |  |
| ROI |  |  | |  | 0.61 | | 0.24 | | **.010** | 0.80 | | | 0.27 | | **.004** |  |
| SG | 0.03 | 0.11 | | .801 | -0.00 | | 0.10 | | .965 | 0.06 | | | 0.11 | | .557 |  |
| Time | 0.00 | 0.01 | | .543 | 0.00 | | 0.01 | | .602 | 0.01 | | | 0.01 | | .352 |  |
| SG x Time | 0.04 | 0.01 | | **< .001** | 0.04 | | 0.01 | | **< .001** | 0.04 | | | 0.01 | | **< .001** |  |
| ROI x SG |  |  | |  | -0.08 | | 0.33 | | .815 | -0.31 | | | 0.37 | | .401 |  |
| ROI x Time |  |  | |  | -0.02 | | 0.02 | | .250 | -0.04 | | | 0.02 | | .075 |  |
| ROI x SG x Time |  |  | |  | -0.01 | | 0.03 | | .715 | 0.01 | | | 0.03 | | .759 |  |
|  |  |  | |  |  | |  | |  |  | | |  | |  |  |
| **Covariate** |  |  | |  |  | |  | |  |  | | |  | |  |  |
| Sex | -0.11 | 0.09 | | .200 | -0.09 | | 0.08 | | .299 | -0.09 | | | 0.08 | | .269 |  |
| **Random Effects** | *SD* | | | | | | | Correlation Intercept | | | | | | | | |
|  | basic | | left.ROI | | | right.ROI | | basic | | | left.ROI | | | right.ROI | | |
| Participant  (Intercept) | 0.40 | | 0.35 | | | 0.35 | |  | | |  | | |  | | |
| Timepoint | 0.01 | | 0.00 | | | 0.00 | | -0.58 | | | -0.46 | | | 0.30 | | |
| Marginal *R^2^* | 0.143 | | | | 0.194 | | | | | | | 0.190 | | | | |
| Conditional *R^2^* | 0.575 | | | | 0.575 | | | | | | | 0.575 | | | | |

*Note*. ROI = region of interest; *SE* = Standard error; *SD* = Standard deviation; SG = stress group.

## References

Bjelland I, Dahl AA, Haug TT, Neckelmann D (2002) The validity of the Hospital Anxiety and Depression Scale. J Psychosom Res 52:69–77.

Cosco TD, Doyle F, Ward M, McGee H (2012) Latent structure of the Hospital Anxiety and Depression Scale: a 10-year systematic review. J Psychosom Res 72:180–184.

Hu L, Bentler PM (1999) Cutoff criteria for fit indexes in covariance structure analysis: conventional criteria versus new alternatives. Struct Equ Modeling 6:1–55.

Rodriguez A, Reise SP, Haviland MG (2016) Evaluating bifactor models: Calculating and interpreting statistical indices. Psychol Methods 21:137–150.

Rosseel Y (2012) lavaan: an *R* package for structural equation modeling. J Stat Soft 48.
